# Parents' Thoughts Regarding Their Normal-Weight Children's Food and Physical Activity as Expressed During Health Conversations With the School Nurse: A Qualitative Analysis Informing Health-Promoting Practices

**DOI:** 10.1177/10598405211025440

**Published:** 2021-06-29

**Authors:** Marianna Moberg, Marie Golsäter, Åsa Norman

**Affiliations:** 1 Department of Global Public Health, 27106 Karolinska Institutet, Stockholm, Sweden; 2 CHILD, School of Health and Welfare, 4161 Jönköping University, Jönköping, Sweden; 3 Futurum—Academy for Health and Care, 369515 Region Jönköping County, Jönköping, Sweden and Linköping University, Linköping, Sweden; 4 Department of Clinical Neuroscience, 27106 Karolinska Institutet, Stockholm, Sweden

**Keywords:** school nurse, parents, parenting, children, health promotion, healthy behaviors, lifestyle, qualitative content analysis

## Abstract

Parents are key to promoting children's healthy growth and development. However, school nurses need knowledge about how to best support parents' health-promoting activities. This study aimed to explore parents' thoughts regarding their normal-weight 6-year-old children's food and physical activity behaviors as expressed during health conversations with the school nurse. Qualitative content analysis of audio-recorded conversations (n = 30) showed that parents think of their children's behaviors in terms of: (a) children's personality in relation to food and physical activity; (b) recognizing children's food and physical activity behaviors; (c) parenting in relation to food and physical activity; (d) interaction with children in situations around food and physical activity; and (e) contextual circumstances to promote children's healthy food and physical activity behaviors. The study contributes with novel knowledge regarding clinical work in health promotion, with suggestions for how school nurses can engage parents in promoting and sustaining healthy food and physical activity behaviors.

## Background

Childhood overweight and obesity is globally one of the major threats to public health, increasing the risk of noncommunicable diseases, and schools comprise important platforms for preventing and detecting risk factors for children's unhealthy weight development (GBD 2015 Risk Factors Collaborators, 2016; World Health Organization, 2016). In Sweden, the prevalence of obesity in the pediatric population has leveled off over the past decades (Eriksson et al., 2018; Moraeus et al., 2014). Nonetheless, inequality between population groups is widening, where a vast increase of prevalence in overweight and obesity is seen in children living in families with lower socioeconomic position (Moraeus et al., 2012, 2014). Moreover, eating and physical activity behaviors established during childhood are likely to track into adolescence and adulthood (Brown et al., 2019; Craigie et al., 2011; Singh et al., 2008). Therefore, interventions focusing on primary prevention of obesogenic risk factors early in life are needed to effectively counteract childhood obesity as it appears harder to influence already established overweight in children (Brown et al., 2019).

Health promotion has been on the global public health agenda for decades, with several charters emphasizing the importance of societies strengthening all individuals' capacity to lead healthy lives as a fundamental human right (Nutbeam, 1998). A systematic and robust approach is fundamental when promoting health in the population, where schools are natural platforms for reaching all children. The World Health Organization (WHO) advocates a whole systems approach to conquer childhood obesity and emphasizes the importance of a preventative approach with health-promoting school environments and provision of guidance for families in making healthy lifestyle choices. Although health systems' responses to conquer childhood obesity differs between countries, WHO stresses the importance of having a robust system in place for monitoring and early detection of risk factors (Baltag et al., 2015; World Health Organization, 2016, 2019).

In Sweden, all children are legally entitled to school health services, including medical, pedagogical, social and psychological efforts that should primarily have a health promoting and disease preventive focus (SFS, 2011). School nurses interact with all children as part of the medical school health services, and as a continuation of the child health care services, where a systematic health-promoting program consisting of regular health visits including health conversations, assessments, and immunizations is followed. The program is free of charge for all children regardless of socioeconomic position and the visits are held at the school facilities. Younger children often visit the school nurse together with a parent or legal guardian and the health visits provide school nurses with an opportunity for monitoring health development and early detection of risk factors (Socialstyrelsen & Skolverket, 2016; Wettergren et al., 2016).

Persistent energy imbalance, that is, energy dense food intake and inadequate physical activity, are modifiable factors contributing to abnormal growth development. In particular, younger children's energy balance is affected by the surrounding environment in a complex manner and the family system has a significant part to play (Niermann et al., 2018). Parents are important influencers for children's behaviors, and are therefore crucial to involve when addressing children's healthy lifestyle behaviors (Golan & Crow, 2004). Previous research describes how school nurses find children's unhealthy behaviors important to identify; at the same time they find addressing the topic in conversations with parents a difficult task (Poutiainen et al., 2015). Further, Swedish school nurses seem to discuss food habits (Golsäter et al., 2016) and physical activity (Golsäter et al., 2015) in health conversations together with older children without parents present. Nonetheless, child health care nurses working with health promotion targeting younger children seldom raise the issue of children's dietary habits and physical activity in conversations with parents during regular health visits (Bohman et al., 2013). This is arguably due to the sensitive topic and fear of harming the relations with parents or children (Regber et al., 2013; Sjunnestrand et al., 2019; Thorstensson et al., 2018). Regarding nurses working with obesity prevention in the school context, Quelly (2014) stresses that nurses' self-efficacy for working with the topic, perceived health benefits for the child, and professional preparedness are facilitating factors for raising the issue with parents.

Primary prevention of known risk factors is strongly recommended as a strategy to defeat childhood obesity (World Health Organization, 2016), for example, intervening before the problem has arisen, such as promoting the emergence of or sustaining healthy habits in children of healthy weight. Nonetheless, there are no studies to our knowledge that explore how parents of normal-weight children view issues regarding food and physical activity behaviors during health visits with school nurses or other primary health professionals. Therefore, understanding how parents of normal-weight children perceive their children's behaviors in health-focused conversations is of importance. This understanding can increase the knowledge regarding what nurses can listen for and use in parents' descriptions to elicit support in creating or maintaining a health-promoting home environment for their children.

### Aim

The aim of the study was to explore parents' thoughts regarding their normal-weight children's food and physical activity behaviors as expressed during health conversations with the school nurse during the first primary school year.

## Methods

### Study Design

This study used real-life audio-recorded health conversations with parents and school nurses from intervention schools participating in the Healthy School Start Plus (HSSP) trial (Elinder et al., 2018). A qualitative explorative inductive design was applied as it was deemed suitable for the research aim (Patton, 2015).

### Population and Data Collection

The school-based cluster-randomized controlled HSSP trial aimed at promoting healthy behaviors and preventing overweight in primary school children through parental support. The 6-month intervention was carried out in 17 suburban primary schools in eight municipalities in mid-Sweden from November 2017 to May 2018 (Elinder et al., 2018). All intervention school nurses (*n* = 7) were trained prior to the intervention in the conversational method, Motivational Interviewing.

In direct proximity to the regular health visit during the first primary school year, school nurses invited parents to an extended health conversation, without the child present. Parents were informed beforehand that focus for the conversation was to discuss their children's lifestyle behaviors. In line with a person-centered approach, school nurses did not follow a set structure for the conversations and parents were encouraged to address areas pressing for their own family situation. As a general introduction to the conversation, with the purpose to spark parents' thoughts and reflections, a topic chart with common areas concerning children's lifestyle behaviors was presented by the school nurses. Examples of areas on the topic chart were screen time, physical activity, fruits and vegetables, sleep, breakfast, snacks and candy, soft drinks, and family meals. School nurses asked explorative questions throughout the conversation to support parents to explore their thoughts regarding the child's behavior, for example, “what are your thoughts when regarding your child's current dietary/physical activity behaviors?” or “If it were to be changed, how would you like it to be instead?”

Health conversations as part of HSSP were conducted at the school nurse's office at the respective schools. All intervention conversations (*N* = 111) were audio-recorded by the school nurse. The sample used for this study comprised of 30 conversations selected from a subset of parents of children with normal weight (*n* = 62) as defined by an international standard (Cole & Lobstein, 2012). The sample was drawn with a maximum variation (Patton, 2015) regarding parent sex, region of birth and education, child sex, school area, and parents' focus for the conversation suitable for the aim, that is concerning food (*n* = 15) and physical activity (*n* = 15). Most conversations were in Swedish (*n* = 26), although four conversations were via a professional interpreter present in the room or via telephone. The length of the sampled conversations ranged between 8 and 39 min with a mean of 18 min.

Sampled conversations concerned children 5–7 years old, with an equal distribution of boys and girls (*n* = 15/15). Twenty parents were born in a non-Nordic^1^ country, and 16 parents had nine years or less of formal education. On two occasions parents came in pairs. Mean age of parents was 38 years, with ages ranging between 28 and 53 years (Table 1).

**Table 1.** Characteristics of Participating Parents, Children, and School Nurses.

Parents (*N* = 32)	
Mothers/fathers (*n*)	18/14
Age (mean years)	38
Born in the Nordic region^a^ (*n*)	12
Highest education: primary school^b^ (*n*)	16
Children (*N* = 30)	
Boys/girls (*n*)	15/15
Age (mean years)	6
Normal weight^c^ (%)	100
School nurses (*N* = 7)	
Female (%)	100
Age (mean years)	47
Years working as school nurse/registered nurse (mean)	3/9

^a^ Including Sweden, Norway, Denmark, Finland, and Iceland.

^b^ Nine years or less of formal education.

^c^ According to Cole and Lobstein (2012).

### Ethical Considerations

All parents provided written consent and the study obtained ethical approval (ref. no. 2017/711-31/1) from the Research Ethic Committee in Stockholm, part of the Swedish Central Ethical Review Board.

### Analysis

The sampled audio recordings were transcribed verbatim and subjected to manifest inductive qualitative content analysis as described by Elo and Kyngäs (2008). The first author (MM) transcribed 18 of the 30 conversations. The remaining 12 sessions were transcribed by an external transcription service. MM listened through all sampled recordings several times and clarified transcripts when needed. Initially, all coauthors (MM, MG, and ÅN) independently read through the same three transcripts and indicated meaning units relevant for the research aim. These were compared in a systematic way to assess the level of abstraction across researchers. Similarities and differences were discussed. Next, all transcripts were read through several times, whereby relevant sections in the transcripts were highlighted through open coding. During written and oral discussions, the data were sorted and grouped under higher headings based on similarities, and by going back and forth between transcripts and codes, preliminary generic categories were formed. The procedure was repeated until the full sample was incorporated and the final categories were agreed. To ensure trustworthiness of the results, authentic and anonymized quotes from the conversations were used to exemplify the categories.

## Results

**Figure 1.**
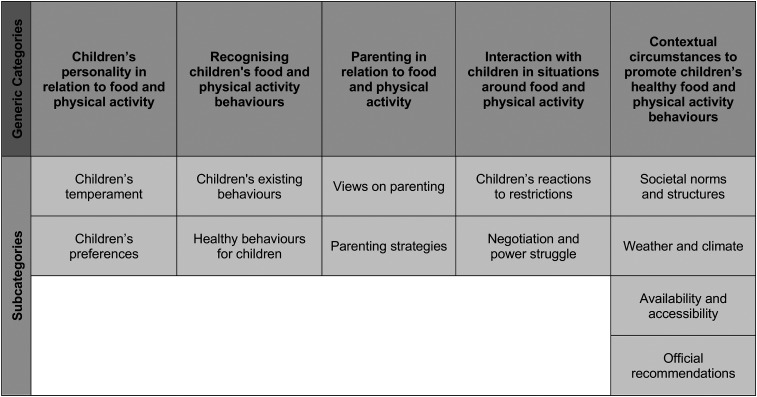
Overview of results. Generic categories and subcategories.

The analysis revealed five generic categories reflecting parents thoughts regarding children's food and physical activity behaviors as expressed in conversations with school nurses: (a) children's personality in relation to food and physical activity, (b) recognizing children's food and physical activity behaviors, (c) parenting in relation to food and physical activity, (d) interaction with children in situations around food and physical activity, and (e) contextual circumstances to promote children's healthy food and physical activity behaviors (Figure 1).

### Children's Personality in Relation to Food and Physical Activity

Parents had thoughts regarding how children's temperament and preferences are expressed and influence food and physical activity.

#### Children's Temperament

Parent's expressed thoughts regarding how children's personality has an impact on their eating and physical activity behaviors and engagement with physical activity and food. Some children were described as assertive when showing what they want, while other children were more timid in their expressions. “Nurse A” asked: “but if you stop feeding him, what do you think will happen?” and “Father 1” answered: “nothing for him, he says nothing. He's hungry, he does not say anything, never.”

Furthermore, parents described how their children's temperament is reflected in their choice of activity, for example, some children choose more physically active games, while others like softer games such as building, drawing and screen play. “Mother 1” describes: “yes when he was a little younger, he constantly just ran, ran … he couldn't sit down, but now it's a little better. But he, when he goes out it's fine. He has energy all the time.”

#### Children's Preferences

Parents described how their children's preferences affect their food and meals. Children were described as having strong preferences about what they want to eat and how the food should be served. Children want the food to be served separated on the plate, refuse to eat breakfast or only want certain kinds of foods and vegetables. “Father 2” expressed: “for example, [if] we have falukorv [sausage], then you can have pasta, or you can have rice … then I think ‘we can make a stroganoff’[sausage stew] … then you hear them just scream ‘noo!’ … they don't like when you mix … and then you think ‘oh well, it did not pass today either’ [laughter].”

### Recognizing Children's Food and Physical Activity Behaviors

In this category, parents expressed thoughts regarding their children's existing eating and physical activity behaviors and whether they wished these were different. Parents also sought to identify healthy behaviors for children and elaborate on their positive or negative consequences.

#### Children's Existing Behaviors

Parents had thoughts regarding current routines for children's eating and physical activity, describing meal orders and level of engagement in sports and outdoor play. Parents expressed a wish for changed behavior, for example, less sedentary time, and a more varied and healthy diet.

Parents also expressed that children's existing behaviors were already good, seeing no room for improvement. They explicitly stated that “everything is fine,” either without any further reflections or at the same time expressing a disguised hesitance and uncertainty about how they can address children's behaviors. This was illustrated by “Mother 2”:

Well, I’m not that fond of it [reducing screen time], I have to say. No… I don't see a major problem in it right now… it works anyway, it's not really that problematic in that sense… If he does his school assignments and it's not a problem, then he may have it [the screen].

#### Healthy Behaviors for Children

Parents suggested what constitute good habits for children and what children's needs are regarding food and physical activity, where vegetable intake, screen time, and consumption of sweets were commonly used as yardsticks. Thoughts about how healthy behaviors are influenced by their own upbringing and experiences of older siblings were expressed, and parents stressed that children's needs are individual and vary with age.

Moreover, parents considered themselves to have a clear idea of benefits and disadvantages of various behaviors considering potential consequences for children's health. Parents expressed thoughts regarding how food choice and eating behaviors affect growth, concentration, and oral health and how screen time can impact sleep and increase sedentary behavior and stress. However, the benefits of using screens for education or even as a motivation for physical activity were also raised. “Mother 3” described:

Because it's not harmful [treadmill], they don't run so they get sweaty. It's just that they’re moving and at the same time they’re looking at the iPad, so that it becomes a compromise. So that they at least get a physical activity, now that it is this time of year [winter] and then it becomes easier to ride a bike and do all sorts of things as well, to go out together and those kinds of things more.

### Parenting in Relation to Food and Physical Activity

Parents expressed thoughts regarding their responsibilities in relation to children's eating and physical activity behaviors. They described their attitudes regarding their role as parents and expressed thoughts regarding parenting and strategies to foster and maintain children's behaviors.

#### Views on Parenting

Parents described their attitudes to the perceived responsibility as a parent as on a sliding scale. On the one hand, parents argued that adults are in charge and responsible for promoting and overseeing healthy behaviors. On the other hand, parents described that their own personal need of solitude at times interferes with upholding routines and restrictions, recognizing that the child's unhealthy behaviors stem from the inconsistency of adults.

Parents also described how children are given informal responsibility to guide the family in matters of food and physical activity, for example, keeping track of and reminding the parent about agreed rules and regulating their own behaviors without guidance from the parent. Children were also described to actively remind the parent of healthy behaviors in the home, advocating for purchasing of healthy food or asking for vegetables missing at the dining table.

In addition, parents conveyed feelings of inadequacy and frustration related to unachieved goals in their child's behaviors. While all parents expressed a will to be involved and do the best for their children's health, many parents described they are feeling stressed and lacking the time and energy to uphold good routines. Failing to implement and maintain these routines while juggling a busy life with work, together with siblings' activities and household tasks, was expressed to cause feelings of inadequacy and guilt.

Yes, there's a bit of problem and so… She has two older sisters who have sports practice almost every night of the week, and I often have to drive them. So, it is hardly ever that we have time to sit like a family and eat… and then I don't know what she eats really and how much she eats. Because usually, I come home and have to throw myself in the car to drive someone… she gets to stay at home with someone who heats food for her, or I heat it quickly and she eats while I drive someone. So, then it becomes a bit that I do not really know if she eats well…Because we will not have this moment where we sit together and eat (“Mother 4”).

Collaboration between parents and shared views on parenting were described as facilitators for children's healthy behaviors. Parents stressed the importance of agreeing on, but also jointly upholding, rules for, for example, screen time or specific days for sweets. In contrast, the feeling of singlehandedly bearing the responsibility was described as an inhibiting factor, for example disagreements on the child's behavior actually being problematic or an unequal workload with children and household duties.

#### Parenting Strategies

Parents described strategies used to influence the child's behaviors. By increasing the availability of healthy options, by being a positive role model, or by encouraging healthy practices, the parent wanted to help establish and maintain the child's healthy behaviors.

Consciously affecting the availability of healthy options for the child was accomplished by parents in several manners. Parents described how healthier food options were made readily available in the home and how rules were introduced to limit unhealthy habits, for example, restricting screen time, serving sweets only on specific occasions, or replacing these by healthier options.

Furthermore, parents described how they, by adapting their own behavior, tried to be a positive role model and influence the child. Examples included engaging in outdoor activities (together with the child), carefully considering what they eat (or not) or avoiding the use of screen devices in the presence of the child. “Father 2” expressed:

I try [to be a role model] anyway, I guess that's what I can be … It's now [at this age] that they are shaped in life so… to set a good example… it's not that easy all the time either… we are all humans of course, but you have to try … it's now they are a bit malleable. It's different when they become teenagers, then it's just “no!” [laughter].

Parents also described how they try to encourage and motivate healthy behaviors in their children by informing them and being responsive to their preferences. Parents gave examples on how they educated the child on the importance of regular food intake and physical activity. Engaging the child in cooking healthy food and encourage the child to try different sports or activities were other examples. Parents also described how meals were adapted to be more appealing to the child, for example plating food in a certain way and buying products similar to those the child enjoy at school.

### Interaction With Children in Situations Around Food and Physical Activity

Parents expressed their thoughts regarding how the parent–child interaction influences situations related to food and physical activity. Parents discussed how children react to parenting strategies and how power struggles between the parent and child are displayed. Parents also described children in social situations and how nonverbal communication occurs in the parent–child interaction around food and physical activity.

#### Children's Reactions to Restrictions

Regarding interaction and restrictions, parents described how children react in different ways when the parent introduces or maintains restrictions around food and physical activity. Descriptions of how children, without disagreements, eat food that is served or turns off the screen when the parent tells them was expressed. In contrast, some children were described to react with anger and frustration when the parent limits the screen time or refused to eat the food served. “Mother 5” expressed: “She gets angry too when you try to take this from her, for example the iPad or so…and it becomes a big problem actually.”

Parents also described situations where children question the parent's decision to limit intake of sweets or screen time, whereby the parent interprets that the child does not understand the purpose of restrictions.

#### Negotiation and Power Struggle

Parents described how negotiation and power struggle manifest in the interaction with children regarding food and physical activity. Screen time is used both as a reprimand for children's nonobedience as well as negotiated reward for finished homework or outdoor play.

He [the child] can borrow the computer sometimes after work. He may have it for a while. We usually trade activity for time. So, if he is are outdoors for half an hour, he gets to have the computer for 15 minutes, or so … (“Mother 6”).

Furthermore, parents described how power struggles occur when the child avoids eating certain foods. In situations like these, the child might cry or say they have a stomach-ache whereas parents try to persuade the child by nagging, forcing, feeding, or acting emotionally hurt.

### Contextual Circumstances to Promote Children's Healthy Food and Physical Activity Behaviors

Parents described how their ability to affect children's behaviors is influenced by the surrounding environment. Social norms, weather and climate, accessibility, and official recommendations all have influence on parents' attitudes and actions in promoting healthy food and physical activity behaviors in their children.

#### Societal Norms and Structures

Parents expressed their thoughts regarding how social expectations influenced the home environment. It was stressed that modern life depends on screens and that even children nowadays use screens instead of other games. In addition, parents discussed how norms among siblings and friends have strong influence on children's choice of activity and that screen use is encouraged and expected from the schools. “Mother 7” expressed:

Yes, but right now it feels like we’re going a bit against that [using screens] … Well… We’re trying to teach them that you shouldn't sit too much with [screens] but almost everything depends on screens … Life depends on it. when the kids get older there is almost no schoolbook, and you ask about something and it is like “search for it on Google.”

#### Weather and Climate

Parents described that the weather and climate affect how much children are physically active. During the Swedish summer, both children and parents are outdoors more often and activate themselves by, for example, cycling or swimming in the lake. When it rains or is cold and dark, like during the winter, children spend less time outdoors and more time at home in front of the screen. This was exemplified in the conversation with “Father 3”:

In Sweden it's a bit cold and a bit dark and you can't always do what you plan. I try to take the opportunity to when the weather [is good], to do good things with the kids. But sometimes it doesn't work because for example you’re thinking of going out to play or doing something outside, but the weather affects.

#### Availability and Accessibility

Parents described how availability and accessibility in the immediate surrounding affect the ability to provide children with healthy food and physical activity. Parents referred to both physical availability in the surrounding area as well as economical accessibility.

Promoting physical activity to children was perceived easy if there was a sense of spare time and were green areas or playgrounds nearby, whereas opportunities for physical activity were impaired when groups for organized physical activity were fully booked or too costly. “Mother 1” expressed: “…um, yes he likes it [swimming class]. But I try to register him but all the time it is full, there is no place.”

#### Official Recommendations

Parents expressed that they were aware of existing recommendations regarding children's food and physical activity. Some recommendations were perceived as hard to accomplish or have unfamiliar examples that parents find difficult to apply in everyday family life. Parents were aware of official labeling of healthy foods and that certain behaviors should be limited (sugar and screen time) while other behaviors are healthy (physical activity, fruits, and vegetables). Parents also described how they used written information as reminders of for example quantities and frequencies.

The kids usually eat sandwich, but now we’re trying to change that… it's not just sandwich they should have; they should also have porridge. Because there is keyhole [The Keyhole symbol^2^], in that. yes, so we also try to change as much as we can (“Mother 8”).

## Discussion

These study results unveil how parents focus the conversation in five specific areas when discussing their normal weight children's food and physical activity behaviors. Parents' thoughts described in the findings can advice school nurses in how to adapt their conversational skills to guide parents regarding: children's personality and preferences, beliefs of what constitutes healthy behaviors for children and possible consequences, thoughts around parents' responsibility and strategies, how parents interact with their children around food and physical activity, and contextual circumstances.

Regarding parents' thoughts on the child as an individual, results indicate that parents sometimes perceive children's food preferences and temperament as barriers to a healthy behavior. Previous studies have shown that children's picky eating can trigger parental emotional responses and practices, and that parent's self-efficacy and beliefs in the child's ability to regulate hunger seem to be modifiable factors for promoting a healthy variation in young children's food preferences (Wolstenholme et al., 2020). Hence, by understanding how parenting practices influence children's food preferences, school nurses can adapt their professional support to needs expressed by the parent in the individual family. In practice, school nurses can provide guidance in conversations with parents for using benign parenting practices that promote healthy behaviors in the child in these situations. Examples could be not to pressure or force a child that refuses to eat certain foods, but rather act as a role model and eat the food him/herself together with the child, and also be persistent in serving a variety of healthy foods to the child (Shloim et al., 2015; Vollmer & Baietto, 2017).

In the present study with parents of normal-weight children, findings reflect that it is rather common for parents to initially state that *everything is fine* regarding their children's behaviors. Nonetheless, a need for support in health promotion, either in changing an unhealthy behavior or in sustaining a healthy behavior over time, may be revealed later on in the conversation. Previous studies have shown that parents consider it to be the responsibility of health care providers to monitor and initiate conversations around children's weight development (Ames et al., 2020) although parents prefer the topic to be addressed sensitively and in a positive manner (Ames et al., 2020). Moreover, sustaining healthy behaviors over time is an important target in public health promotion. Regarding promoting healthy behaviors to young children, a family- and person-centered approach can support nurses to raise topics related to children's behaviors by focusing on parent's wishes and capability (Kitson et al., 2013). Nonetheless, the practical work on how to support or promote healthy behaviors has rendered little attention in clinical training. To further add to the complexity for school nurses, working with health promotion often includes working with families before problems arise, where parents may not perceive a problematic situation or behavior and need more support. Thus, specific conversational skills from the nurses to evoke reflections regarding the need to sustain healthy behaviors over time might be required, something nurses are seldom trained in. Person-centered care is considered a core competence for health care professionals both globally (Bartz, 2010) and in Sweden (Svensk sjuksköterskeförening et al., 2019) emphasizing patient involvement and respectful patient–provider relationship (Kitson et al., 2013). School nurses engaging in health promotion through conversations with parents need to be skilled in person-centered conversational techniques that support parents in working with either small changes or sustaining healthy behaviors over time. For this purpose, the person-centered method Motivational Interviewing (Miller & Rollnick, 2012), developed for supporting behavioral change and sustainment of healthy behaviors, can be useful (Borrelli et al., 2015). In addition, previous research show that nurses perceive Motivational Interviewing as a useful method when meeting parents in conversations regarding lifestyle behaviors (Bonde et al., 2014; Söderlund et al., 2010).

Regarding parents' attitudes and practices, previous studies have shown that certain parenting practices, for example, ensuring availability and acting as role models are effective when promoting children's healthy eating (Vollmer & Mobley, 2013) and physical activity (Hutchens & Lee, 2017). Parents perceptions and behaviors are influenced by a complex web of factors, ranging from personal to societal aspects (Pocock et al., 2010). In the present study, parents described confidence in strategies such as child involvement and parental role modeling. Nonetheless, parents also expressed insecurity in how to encourage the child to change an established behavior, whereas some parents described how they apply unfavorable strategies such as restrictions, nagging, or blaming to persuade the child to a desired behavior. Available evidence indicates that parents using foods as reward, pressuring the child to eat, and restricting foods seem to influence children to prefer unhealthy foods more often (Vollmer & Baietto, 2017). Conversely, engaging the child in preparing the food, ensuring availability of healthy foods in the home, acting as a role model, and explaining benefits to the child seem to be associated with children preferring health foods (Vollmer & Baietto, 2017). Therefore, school nurses need to have knowledge regarding what parenting practices to encourage to guide parents in this regard when discussing children's healthy behaviors and provide this information in a person-centered manner in health conversations.

Regarding interaction between the parents and the child, parents in our study described how applied strategies turn out, and how children respond either with ease and agitation, in situations around food and physical activity. Norman et al. (2018) argue that parents who trust and are responsive to their child's abilities and preferences, and recognize a higher sense of responsibility as parents, interact and guide their child in a more constructive manner (Norman et al., 2018). Similarly, previous research concludes that parents with an *authoritative* parenting style*,* which includes clear boundaries and expectations within an atmosphere with emotional warmth, generally seem to have a protective effect on children's healthy lifestyle and weight development. Whereas parents with an *authoritarian* parenting style, applying harsh practices ignoring the child's preferences, or *uninvolved* and *indulgent* parenting styles, more often have children with unhealthy behaviors (Shloim et al., 2015; Vollmer & Mobley, 2013). School nurses engaging in conversations with parents around parent–child interactions can benefit from being aware of benign parenting styles and associated parenting practices (Darling & Steinberg, 1993), to guide parents in their roles when applying boundaries with warmth in order to promote healthy behaviors.

Regarding contextual circumstances related to children's healthy behaviors, parents in our study expressed that their efforts to promote healthy behaviors in their children often are influenced by the surrounding context, and social norms and structures are raised as both barriers and facilitators. Similarly, previous research has concluded that siblings and peers play a vital role in children's food behaviors (Ragelienė & Grønhøj, 2020) and physical activity (Kracht & Sisson, 2018; Maturo & Cunningham, 2013). Furthermore, parents struggle to promote healthy behaviors when their young children frequently are exposed to unhealthy foods and sedentary temptations in their close environment (Stenhammar et al., 2012). Regarding the built environment, associations between childhood obesity and availability of parks and playgrounds in a neighborhood is strong and accentuated by socioeconomic position (Morgan Hughey et al., 2017). In addition, parents perceive safety in the surrounding environment a principal supportive factor for children's active transportation to school (Aranda-Balboa et al., 2019) and out-door play (Carver et al., 2008). Furthermore, parents in our study express that official recommendations regarding children's healthy food and physical behaviors sometimes are difficult to apply, whereas availability of economically accessible organized activities was expressed as barriers. Previous studies indicate that lack of time and money are crucial aspects in this (Stenhammar et al., 2007) and that parents request practical and guiding information such as brochures and websites to feel confident in addressing the issue in everyday life (Ames et al., 2020). Written information, such as brochures and webpages, could not only aid school nurses in communicating evidence-based practices to parents, but also act as reminders for parents in the home environment. Furthermore, our findings regarding contextual circumstances and children's healthy behaviors confirm the notion that a whole systems approach is needed to address childhood overweight and obesity (World Health Organization, 2016), where local governments, schools, and parents should cooperate regarding these contextual factors. School nurses need to understand parent's perceptions regarding contextual barriers to encourage parents to engage in facilitating interventions such as involving children's friends and arranging safe and active cotransportation with other parents. Furthermore, it is of importance for school nurses to have updated knowledge of locally provided activities as well as written information, digital and physical, regarding recommendations and activities for children to offer parents when requested.

### Strengths and Limitations

The strengths and limitations of this study were related to study design and analysis. Regarding data collection, this consisted of audio-recorded real-life health conversations with parents, conducted and recorded by the school nurses. On the one hand, this might have posed a risk for loss of valuable information when the researcher was not able to ask clarifying questions, or if parts of the conversation were left out of the recording. On the other hand, this method was a strength, providing unobtrusive and objective information on parent's thoughts, also leaving an audit trail to further strengthen trustworthiness. Regarding sampling, a strength was the maximum variation sampling that ensured a diversity of parents' thoughts, and thus the sampled conversations displayed a broad variation in preunderstanding and focus. Nonetheless, a limitation could be that all parents had received information included in the HSSP trial, which in turn could have affected their thoughts and thereby the results of this study. Regarding analysis and interpretation of findings, strengths comprised of all involved coauthors engaging in continuous discussions throughout the research process. Furthermore, coauthors are experienced in the school nurse setting, and in the delicate work of translating qualitative research from Swedish to English. Insights from this study may be applicable to similar contexts involving nurses working with health promotion, targeting parents and young children. Nonetheless, transferability of qualitative research results is ultimately interpreted by the reader based on a rigorous description of the study context (Lincoln & Guba, 1985).

## Conclusions

Results of this study can guide school nurses on what main areas to be susceptible for in health-promoting conversations with parents around children's food and physical activity behaviors. The results might also suggest what further competence, training and conversational skill sets nurses need to acquire to best support parents in health-promoting conversations and what tools are needed to stimulate healthy lifestyle environments for young children.

### Future Research

Future research should focus on effective skill development for school nurses meeting parents in health-promoting situations, with the objective to sustain already healthy behaviors in a healthy child population, to prevent unhealthy growth development.

## Notes

1. The *Nordic countries* (Swe: *Norden*) are a geographical region and include the sovereign states of Sweden, Norway, Denmark, Iceland, and Finland.

2. The Keyhole symbol (Swe: *Nyckelhålet*) is a food label from the Swedish National Food Agency with the purpose to guide consumers identify healthier food options.
